# Randomized clinical trial of negative pressure wound therapy as an adjunctive treatment for small‐area thermal burns in children

**DOI:** 10.1002/bjs.11993

**Published:** 2020-09-14

**Authors:** C. C. Frear, L. Cuttle, S. M. McPhail, M. D. Chatfield, R. M. Kimble, B. R. Griffin

**Affiliations:** ^1^ Centre for Children's Burns and Trauma Research South Brisbane Australia; ^2^ Queensland Children's Hospital South Brisbane Australia; ^3^ Faculty of Medicine University of Queensland Herston Australia; ^4^ School of Biomedical Sciences Brisbane Queensland Australia; ^5^ Australian Centre for Health Services Innovation and Centre for Healthcare Transformation School of Public Health and Social Work Brisbane Queensland Australia; ^6^ School of Nursing, Faculty of Health Queensland University of Technology Brisbane Queensland Australia; ^7^ Clinical Informatics Directorate, Metro South Health Brisbane Queensland Australia

## Abstract

**Background:**

The efficacy of negative pressure wound therapy (NPWT) in the acute management of burns remains unclear. The purpose of this trial was to compare standard Acticoat™ and Mepitel™ dressings with combined Acticoat™, Mepitel™ and continuous NPWT to determine the effect of adjunctive NPWT on re‐epithelialization in paediatric burns.

**Methods:**

This two‐arm, single‐centre RCT recruited children with acute thermal burns covering less than 5 per cent of their total body surface area. The primary outcome was time to re‐epithelialization. Blinded assessments were performed using photographs captured every 3–5 days until discharge. Secondary measures included pain, itch, grafting, perfusion and scar management referrals.

**Results:**

Some 114 patients were randomized. Median time to re‐epithelialization was 8 (i.q.r. 7–11) days in the NPWT group and 10 (8–14) days in the control group. In a multivariable model, NPWT decreased the expected time to wound closure by 22 (95 per cent c.i. 7 to 34) per cent (*P* = 0·005). The risk of referral to scar management was reduced by 60 (18 to 81) per cent (*P* = 0·013). Four participants in the control group and one in the NPWT group underwent grafting. There were no statistically significant differences between groups in pain, itch or laser Doppler measures of perfusion. Adverse events were rare and minor, although NPWT carried a moderate treatment burden, with ten patients discontinuing early.

**Conclusion:**

Adjunctive NPWT hastened re‐epithelialization in small‐area burn injuries in children, but had a greater treatment burden than standard dressings alone. Registration number: ACTRN12618000256279 ( http://ANZCTR.org.au).

## Graphical abstract

In this RCT, children with small‐area thermal burns were randomized to standard silver‐impregnated dressings or standard dressings in conjunction with continuous negative pressure wound therapy (NPWT). Adjunctive NPWT decreased time to re‐epithelialization, with attendant improvements in dressing change requirements and scar management referrals. There were no statistically significant differences in pain, itch, or laser Doppler measures of perfusion.

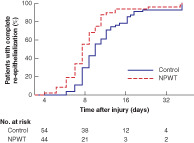

Negative pressure wound therapy hastens epithelialization

## Abstract (es)

**Antecedentes:**

La eficacia del tratamiento de las heridas con presión negativa (*negative pressure wound therapy*, NPWT) en el tratamiento agudo de las quemaduras sigue sin estar claro. El propósito de este ensayo clínico fue comparar los apósitos estándar del tipo Acticoat™ y Mepitel™ con la combinación de Acticoat™, Mepitel™ y NPWT continua para determinar el efecto de la adición de NPWT en la reepitelización de las quemaduras en pediatría.

**Métodos:**

Ensayo controlado y aleatorizado, con dos brazos y unicéntrico, que reclutó niños con quemaduras térmicas agudas que afectaban < 5% de la superficie corporal total. El resultado primario fue el tiempo hasta la reepitelización. Se realizaron evaluaciones a ciegas utilizando fotografías tomadas cada 3‐5 días hasta el alta hospitalaria. Las medidas secundarias incluían dolor, picor, injerto, perfusión y derivación para el tratamiento de las cicatrices.

**Resultados:**

Se aleatorizaron un total de 114 pacientes. La mediana de tiempo hasta la reepitelización fue 8 días (rango intercuartílico, *interquartile range*, IQR 7‐11) en el grupo NPWT y 10 días (8‐14) en el grupo control. En el modelo multivariable, el uso de NPWT disminuyó los días previstos hasta el cierre de la herida en un 22% (i.c. del 95% 7‐34%; *P* = 0,005). El riesgo de ser derivado para el tratamiento de la cicatriz se redujo en un 60% (18‐81%; *P* = 0,013). Cuatro participantes en el grupo control y uno en el grupo NPWT fueron sometidos a injertos. No hubo diferencias estadísticamente significativas en el dolor, picor, o mediciones de la perfusión con Doppler laser. Los eventos adversos fueron raros y menores, aunque NPWT conllevó una carga de tratamiento moderada con 10 pacientes que lo suspendieron precozmente.

**Conclusión:**

El tratamiento complementario de la herida con presión negativa acelera el tiempo hasta la reepitelización en quemaduras de pequeña extensión en niños, pero implica una mayor carga de tratamiento.

## Introduction

Small‐area burns remain a common form of childhood injury, contributing substantially to the non‐fatal burden of disease^1^. Although typically handled on an outpatient basis, these burns nevertheless demand careful treatment to ward off infection and optimize healing^2^. Failure to achieve prompt wound closure significantly increases the risk of hypertrophic scar formation^3^. Affecting between 16 and 35 per cent of children after burn injury^4^, ^5^, ^6^, ^7^, ^8^, scarring carries the potential to have a detrimental effect on long‐term physical, cosmetic and psychological outcomes.

Negative pressure wound therapy (NPWT) is a non‐invasive treatment that has shown promise as a facilitator of burn wound healing^9^, ^10^, ^11^. Nonetheless, the body of rigorous evidence from RCTs has not been commensurate with its growing popularity^12^, ^13^, ^14^. Recently, three small RCTs^15^, ^16^, ^17^ reported significant improvements with NPWT in re‐epithelialization, histological and biochemical markers of wound healing, skin grafting, bacterial infection and scarring. These trials, however, were constrained by their reliance on suboptimal treatment comparators and unvalidated surrogate outcomes. The aim of this research was to determine the efficacy of NPWT as an adjunct to standard silver‐impregnated dressings, with a focus on patient‐centred outcomes in children with small‐area burns.

## Methods

SONATA in C (Study Of Negative pressure wound therapy as an Adjunctive Treatment for Acute burns in Children) is a single‐centre, two‐arm, parallel, randomized, active‐controlled trial. The trial protocol received approval from the Children's Health Queensland Hospital and Health Service and University of Queensland Human Research Ethics Committees (HREC/17/QRCH/279, SSA/17/QRCH/292, 2018000335/HREC/17/QRCH/279). The methodology has been published previously^18^ and the trial was registered with the Australian New Zealand Clinical Trials Registry before the start of recruitment (ACTRN12618000256279).

### Setting and recruitment

Participants were recruited from a large tertiary‐level metropolitan children's hospital. Children aged less than 17 years were eligible if they presented with a thermal burn covering less than 5 per cent of their total body surface area (TBSA). Patients were excluded if the burn injury occurred more than 7 days before presentation, affected the face, or was expected by clinicians to achieve full re‐epithelialization with standard care before the next dressing change 3–5 days later.

### Procedures and interventions

Participants were randomized to receive either standard dressings (control group) or standard dressings in combination with NPWT (NPWT group). The standard dressings consisted of Acticoat™ (Smith & Nephew, Hull, UK), a nanocrystalline silver‐impregnated fibre mesh, and Mepitel™ (Mölnlycke Healthcare, Mikkeli, Finland), a silicone interface. The intervention was delivered via a RENASYS TOUCH™ vacuum pump (Smith & Nephew) set to a continuous subatmospheric pressure of 80 mmHg. For burns involving the extremities in children aged less than 12 months, a pressure of 40 mmHg was employed instead to reduce the risk of ischaemic damage. Participants returned to the burn centre every 3–5 days for removal and reapplication of NPWT and/or standard dressings until wound closure.

### Baseline characteristics

Investigators not involved in the care of participants were responsible for trial recruitment, allocation and data collection. They recorded the following baseline characteristics for all eligible patients: age, sex, Fitzpatrick skin type, time of injury, wound aetiology, anatomical location, previous wound care, and clinical assessments of depth and TBSA.

### Primary outcome measure

The primary outcome was the time elapsed, in days, from the point of injury to re‐epithelialization of the wound. Photographs of wounds captured during dressing changes underwent blinded review by a panel of three experienced burn clinicians, who indicated for each participant the dressing change at which the wounds were at least 95 per cent re‐epithelialized. The assessments favoured by at least two of the blinded reviewers were used in the analysis. In two instances where there was no majority, members of the panel convened with a fourth burn clinician to reach a consensus.

### Secondary outcome measures

#### *Pain and itch*

At each dressing change, investigators recorded preprocedural and postprocedural pain and itch, as well as retrospective measures of peak procedural pain. Caregivers were also prompted to document pain and itch at home by means of an electronic survey 24 h after each dressing change.

Observational measures were obtained from clinicians and caregivers. Clinicians evaluated pain and distress in clinic via the Face, Legs, Activity, Cry, Consolability scale^19^. Caregivers used an 11‐point (0–10) numerical rating scale (NRS) to evaluate pain, and the Toronto Paediatric Itch Scale^20^ to assess itch in participants under 5 years of age. Self‐reports of pain were obtained using the Revised Faces Pain Scale^21^ in children aged 4–7 years, and the NRS in those aged 8 years and older. Participants older than 5 years self‐reported itch by means of the Itch Man Scale^22^.

In accordance with the burn centre's standard practice, pharmacological analgesia on initial presentation and first dressing change consisted of a combination of oxycodone (0·1–0·2 mg/kg), paracetamol (15 mg/kg) and/or ibuprofen (10 mg/kg). Participants with high levels of pain were also provided with nitrous oxide/oxygen (Entonox™; BOC Healthcare, Manchester, UK).

#### *Wound perfusion*

Laser Doppler imaging (LDI) was performed to assess mean flux of the defined wound area using a MoorLDI2‐Burn Imager™ (Moor Instruments, Axminster, UK) at the baseline visit and first dressing change^23^.

#### *Skin grafting, adverse events and scar management*

Investigators noted all cases of skin grafting, adverse events and referrals to scar management. Patients were referred to scar management if re‐epithelialization took 14 days or more^6^, they received a graft, or were judged by clinicians to be at high risk of functionally or cosmetically impactful scar formation.

#### *Management, movement and satisfaction with treatment*

At every visit, clinicians were asked to rate the ease of applying and removing dressings on an 11‐point NRS. Caregivers and children aged 8 years and older assessed the impact on their ease of movement, again using an 11‐point NRS. The original protocol called for a concurrent measure of treatment satisfaction. This was abandoned at an early stage, however, as a result of most families' self‐acknowledged lack of familiarity with burn management. Therefore, the scale was modified to assess the ease of managing the dressings at home.

#### *Scarring*

Participants were invited to attend follow‐up appointments 3 and 6 months after injury to evaluate between‐group differences in hypertrophic scarring. Ultrasound imaging and colorimetry were performed at both the burn site and a comparator region of unaffected skin on the contralateral side (or the nearest region of unaffected skin). Images captured using a BT12 Venue 40 MSK ultrasound machine (General Electric, Little Chalfont, UK) were evaluated by a blinded reviewer, who measured the distance between the outer border of the epidermis and the inner border of the dermis. Colorimetry was undertaken using a DSM II ColorMeter® (Cortex Technology, Hadsund, Denmark), with three readings recorded per site of pigmentation (L) and erythema (a). Caregivers and/or children completed the patient component of the Patient and Observer Scar Assessment Scale (POSAS)^24^ and Brisbane Burn Scar Impact Profile (BBSIP)^25^ to provide subjective assessments of scar severity and burn‐specific health‐related quality of life respectively. Families who declined to attend the appointments were instead sent online POSAS and BBSIP questionnaires.

### Randomization

An unpredictable 1 : 1 allocation sequence was generated by a statistician^26^. Randomization was stratified by age (0–3, 4–7, 8–16 years), and a permutated block method with random block sizes of 4 and 6 was used. The sequence was uploaded to the REDCap randomization module, which concealed allocation until the point of randomization.

### Blinding

Blinding was not possible for participants and treating clinicians because of obvious differences in physical appearance between the interventions. However, the primary outcome was evaluated by blinded review. Two of the reviewers were involved in the care of at least one of the trial participants. To reduce the possibility of recognition, the images were edited to remove all potentially identifying attributes.

### Statistical analysis

The sample size was calculated from the primary outcome using an independent‐samples *t* test. A previous study^27^ reported a mean(s.d.) time to re‐epithelialization of 12·4(5·4) days with Acticoat™ therapy. Given the finding of Deitch and colleagues^3^ that hypertrophic scarring was absent in patients whose burn injury healed in less than 10 days, a mean time to re‐epithelialization of 9 days for the NPWT group was viewed as a minimum clinically important improvement. With more than 80 per cent power and a significance level of 0·05, and assuming an attrition rate of 20 per cent, it was determined that a minimum enrolment of 104 participants was required. No interim analyses were performed.

Between‐group differences in demographic and baseline data were assessed using the Student *t* test and Mann–Whitney *U* test for continuous variables, and the χ^2^ test and Fisher's exact test for categorical variables.

**Fig. 1:**
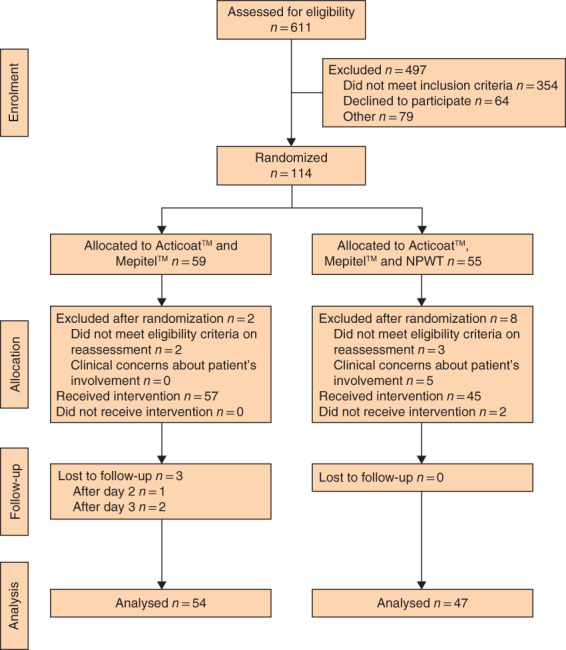
CONSORT diagram for the trial

Time to re‐epithelialization was analysed by negative binomial regression^28^, ^29^. The ratio of means (NPWT *versus* control group) was reported using the incidence rate ratio (IRR) with 95 per cent confidence interval. The primary analysis sought to incorporate participants who underwent split‐thickness skin grafting. Because the time of grafting is often influenced by factors unrelated to the wound, such as operating room availability, a previously described method^30^ was employed to estimate spontaneous time to re‐epithelialization using a dummy value of 1 day greater than the longest duration of wound closure documented for any non‐grafted wound. The wound with the greatest re‐epithelialization time (40 days) had the same depth as the grafted burns. A sensitivity analysis excluding all patients with grafted wounds was also performed. Further sensitivity analyses were undertaken using Kaplan–Meier and Cox proportional hazards models, with estimation of hazard ratios and 95 per cent confidence intervals. A *post hoc* subgroup analysis was conducted assessing re‐epithelialization in participants treated within a 48‐h time frame *versus* those who presented later. A subgroup × NPWT interaction term and a main effect for subgroup were added to the Cox model. A modified Poisson regression model was used to assess the need for scar management. Pain and itch were evaluated via multilevel generalized linear mixed‐effects models with a log‐link function and Poisson distribution, with preprocedural pain at first presentation entered as a co‐variable in pain analyses.

Prespecified demographic and injury‐related variables (time to treatment, depth, TBSA, aetiology, anatomical location and skin type) were tested against primary and secondary outcomes. Any variables with *P* ≤ 0·100 in univariable analyses were included as co‐variables in the multivariable analyses. Significance was set at *P* < 0·050. Analyses were conducted using SPSS® version 25 (IBM, Armonk, New York, USA) and Stata® version 16 (StataCorp, College Station, Texas, USA).

## Results

From May 2018 to January 2019, 114 patients were randomized (*Fig*. *1*). Demographic and injury characteristics were balanced across the two groups (*Table* *1*).

**Fig. 2:**
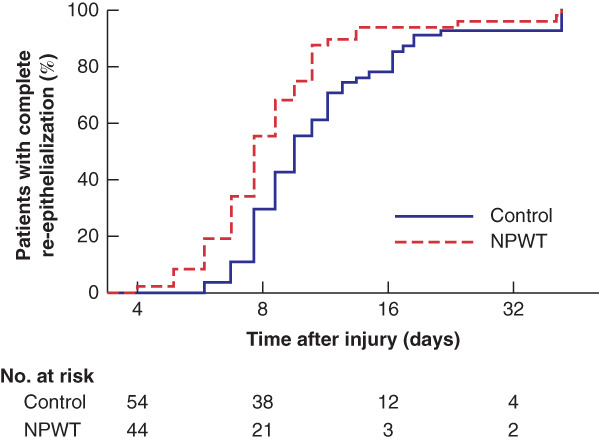
Kaplan–Meier analysis of time to re‐epithelialization by treatment group NPWT, negative pressure wound therapy.

**Table 1:** Demographic and baseline clinical characteristics

	Control (*n* = 54)‡	NPWT (*n* = 47)
**Age (years)** *	4 (1–9)	4 (1–8)
< 8	35 (65)	31 (66)
**Sex ratio (M : F)**	31 : 23	28 : 19
**Total body surface area affected (%)** *	1 (1–2)	1·5 (1–2)
**Depth**		
Superficial partial thickness	36 (67)	32 (68)
Deep partial thickness	18 (33)	15 (32)
**Baseline perfusion (PU)** †, §	846(286)	866(252)
**Mechanism of burn**		
Scald	35 (65)	28 (60)
Contact	18 (33)	17 (36)
Flame	1 (2)	2 (4)
**Time to presentation (days)** *	3 (2–3)	3 (1–4)
**Presentation within 48 h of injury**	18 (33)	16 (34)
**Adequate first aid provided**	43 (80)	38 (81)
**Anatomical location**		
Upper limb(s)	17 (31)	21 (45)
Lower limb(s)	21 (39)	15 (32)
Chest, torso, back	12 (22)	8 (17)
Genitals, buttocks	2 (4)	2 (4)
Multiple sites	2 (4)	1 (2)
**Fitzpatrick skin type**		
1	3 (6)	5 (11)
2	17 (31)	18 (38)
3	20 (37)	16 (34)
4	11 (20)	6 (13)
5	1 (2)	1 (2)
6	2 (4)	1 (2)

Values in parentheses are percentages unless indicated otherwise; values are

* median (i.q.r.) and

† mean(s.d.).

‡ Excluding three participants who were lost to follow‐up.

§ Data collected from 40 participants (74 per cent) in control group and 32 (68 per cent) in negative pressure wound therapy (NPWT) group. PU, perfusion units.

Two participants in the control group and three in the NPWT group were excluded after randomization owing to either a modification of their history or a reassessment indicating ineligibility. A further five participants in the NPWT group were excluded because of concerns regarding baseline anxiety levels or social circumstances (such as lack of easy access to assistance in the event of device malfunction). The decision was made by treating clinicians to exclude these participants from the trial altogether. All had superficial partial‐thickness burns with a TBSA of 2 per cent or less.

The intervention was administered for a minimum of 2 days in 45 of 47 children in the NPWT group. Of these, 31 participants received the intervention until wound closure. Among the remaining 14, the median duration of NPWT was 3 (i.q.r. 3–4) days. It was discontinued in four children for clinical reasons. In the other ten, participants or caregivers declined reapplication of NPWT at the first or second dressing change.

### Time to re‐epithelialization

A total of 96 of 101 participants (95·0 per cent) achieved full re‐epithelialization spontaneously. There was good agreement among the blinded assessors (intraclass correlation coefficient 0·85). The median time to re‐epithelialization was 10 (i.q.r. 8–14) days in the control group and 8 (7–11) days in the NPWT group. After adjusting for depth, anatomical location and aetiology, NPWT reduced the expected time to re‐epithelialization by 22 per cent (IRR 0·78, 95 per cent c.i. 0·66 to 0·93; *P* = 0·005). Kaplan–Meier curves for re‐epithelialization time in the NPWT and control groups are shown in *Fig*. *2*. In a multivariable Cox regression model adjusted for depth and anatomical location, the hazard ratio for the NPWT group was 1·66 (95 per cent c.i. 1·11 to 2·47; *P* = 0·014).

A sensitivity analysis excluding patients who received grafts yielded consistent findings (IRR 0·85, 95 per cent c.i. 0·73 to 0·98; *P* = 0·027). Median time to wound closure remained unchanged in both groups. A *post hoc* subgroup analysis investigating whether NPWT exhibited greater efficacy if applied within 48 h of the burn was inconclusive (*Table* *2*). The hazard ratio for the subgroup in which the NPWT was applied within 48 h was 1·54 (95 per cent c.i. 0·64 to 3·62) times that for the subgroup with a longer time to treatment (*P* = 0·330).

**Table 2:** Subgroup analysis of time to re‐epithelialization

	Time to re‐epithelialization (days)*				
Time to presentation (h)	Control	NPWT	Hazard ratio†, ‡	*P*	*P* for interaction
< 48	9 (8–13·75) (*n* = 18)	7·5 (5·25–8) (*n* = 16)	2·26 (1·11, 4·61)	0·025	0·330
≥ 48	10·5 (9–14·75) (*n* = 36)	9 (7–11) (*n* = 31)	1·47 (0·90, 2·40)	0·121

Values are

* median (i.q.r.);

† values in parentheses are 95 per cent confidence intervals. NPWT, negative pressure wound therapy.

‡ Cox proportional hazards analysis adjusted for depth and anatomical location.

### Pain and itch

There were no statistically significant differences between treatment groups in pain or itch (*Table* *3*; *Table* *S1*, supporting information).

**Table 3:** Mixed‐effects modelling for pain and itch measures

	Ratio of mean score for NPWT/control	*P*
**During dressing application/removal**		
FLACC scale	1·09 (0·83, 1·43)	0·534
Observational NRS	1·02 (0·68, 1·54)	0·917
Self‐reported NRS	1·74 (0·95, 3·19)	0·072
**After dressing application/removal**		
Observational NRS	1·28 (0·64, 2·58)	0·484
Self‐reported NRS	1·36 (0·57, 3·25)	0·485
Toronto Pediatric Itch Scale	1·19 (0·29, 4·85)	0·808
Itch Man Scale	1·21 (0·49, 2·99)	0·679
**At home**		
Observational NRS	0·86 (0·52, 1·41)	0·554
Self‐reported NRS	1·05 (0·55, 2·01)	0·874
Toronto Pediatric Itch Scale	0·79 (0·40, 1·56)	0·499
Itch Man Scale	1·39 (0·95, 2·05)	0·092

Values in parentheses are 95 per cent confidence intervals. Data from the first two clinical visits and home questionnaires were used in the analysis. Only 17 children fell within the 4–7‐year age range, too few to allow a proper analysis of Revised Faces Pain Scale data. Additionally, three participants in the control group and one in the negative pressure wound therapy (NPWT) group had a dressing change under general anaesthesia; it was not possible to collect pain or itch measures for these procedures. FLACC, Face, Legs, Activity, Cry, Consolability; NRS, numerical rating scale.

### Wound perfusion

LDI was performed successfully in 75 of 104 participants (72·1 per cent) at the baseline visit, 64 (61·5 per cent) at the first dressing change, and 50 (48·1 per cent) at both time points. At the first change of dressings, mean perfusion was higher in the NPWT group than in the control group (mean(s.d.) 981(296) *versus* 882(250) perfusion units (PU); *P* = 0·153). In a separate exploratory analysis, the wound area of children who received NPWT within 48 h of injury exhibited higher mean perfusion at 3–5 days after the burn compared with the remainder of the cohort (1085(294) *versus* 951(254) PU; *P* = 0·104).

### Grafting

Split‐thickness skin grafting was undertaken in four participants (7 per cent) in the control group and one (2 per cent) in the NPWT group.

### Scar management referrals

A statistically higher proportion of children in the control group received referrals to scar management than in the NPWT group: 15 of 54 (28 per cent) *versus* five of 47 (11 per cent) (*P* = 0·031). After adjusting for age and depth, the risk of referral to scar management was reduced by 60 per cent in the NPWT group (relative risk 0·40, 95 per cent c.i. 0·19 to 0·82; *P* = 0·013).

### Dressing changes

The median number of dressing changes until wound closure was 2 (i.q.r. 2–3) in the NPWT group and 3 (2–4) in the control group (*P* = 0·003).

### Scarring

Among the 96 participants whose wound re‐epithelialized spontaneously, scar questionnaires were completed by 67 (70 per cent) at 3 months and 58 (60 per cent) at 6 months, with 53 (55 per cent) participating at both time points. Most were submitted online, as rates of attendance at in‐person follow‐up visits were low: 28 children (29 per cent) presented at 3 months, 23 (24 per cent) at 6 months and 14 (15 per cent) at both time points. The questionnaires showed that caregiver perception of scar severity was modestly improved in the NPWT group, although these improvements were not statistically significant (*Table* *S2*, supporting information). No statistically significant differences were detected at either time point in burn‐specific health‐related quality of life (*Tables* *S3* and *S4*, supporting information) or colorimetry (*Table* *S5*, supporting information). The median relative difference in scar thickness was statistically lower in the NPWT group at 3 months (*P* = 0·018), but not at 6 months (*P* = 0·928) (*Table* *S5*, supporting information).

### Movement and management

At the first dressing change, caregivers rated movement and management as more difficult in the NPWT group for participants aged less than 8 years (*P* < 0·001) (*Table* *4*). The intervention was also rated as more difficult to manage by clinicians (*P* < 0·001), taking longer to apply (*P =* 0·002) and remove (*P* < 0·001). Self‐reports by older children, however, revealed no statistically significant between‐group differences in management (*P* = 0·116) or movement (*P* = 0·061).

**Table 4:** Assessments of ease of management and movement

	Control	NPWT	*P* *
**Initial visit**			
Ease of application	8 (7–10) (*n =* 54)	7 (5–8) (*n =* 45)	< 0·001
Duration of application (min)	11 (7–15·75) (*n =* 56)	16 (10–19) (*n =* 47)	0·002
**Second visit**			
Ease of removal	9 (8–10) (*n =* 50)	7 (6–8) (*n =* 41)	< 0·001
Duration of removal (min)	5 (4–8) (*n =* 53)	8 (6–10·5) (*n =* 45)	< 0·001
**Ease of management at home**			
Caregiver report (child <8 years)	10 (9–10) (*n =* 35)	7·5 (5–10) (*n =* 30)	< 0·001
Child self‐report (≥ 8 years)	10 (8–10) (*n =* 19)	8·5 (6·25–9) (*n =* 16)	0·116
**Ease of movement**			
Caregiver report (child <8 years)	10 (8–10) (*n =* 35)	6·5 (4–8) (*n =* 30)	< 0·001
Child self‐report (≥ 8 years)	10 (6–10) (*n =* 19)	7 (5–8) (*n =* 16)	0·061

Values are median (i.q.r.); unless indicated otherwise, values are scores on an 11‐point Likert scale (0, extremely difficult to manage/move; 10, extremely easy to manage/move). NPWT, negative pressure wound therapy.

* Mann–Whitney *U* test.

### Adverse events

NPWT was discontinued in four patients for clinical reasons: wound maceration (1), periwound blistering (a skin reaction to the adhesive film; 1) and exacerbation of pre‐existing viral illnesses unrelated to burns (2). Blistering was observed in an additional two participants whose burns were fully re‐epithelialized and did not require further care. There were no instances of wound infection. In the control group, there was a single case of exacerbation of a pre‐existing viral disease, but no other adverse events.

### Treatment burden

Ten families in the intervention group requested discontinuation of NPWT before the primary endpoint had been reached. Among the reasons provided for declining reapplication, caregivers listed the physical burden of the pump, mechanical issues and difficulties attending school. Three families presented to the emergency department with technical problems (such as air leaks and charging abnormalities) they could not resolve at home.

## Discussion

NPWT accelerated re‐epithelialization in children with partial‐thickness thermal burns of less than 5 per cent TBSA. The decrease in expected time to wound closure by 22 per cent corresponded to a reduction in the number of dressing changes required, and a 60 per cent decline in the risk of referral for scar management. Prompt wound healing represents one of the foremost challenges in the management of small‐area thermal injuries^2^, which comprise the majority of cases treated by paediatric burn services^31^, ^32^, ^33^. Delays in healing lead to extended resource‐ and time‐intensive care, and increase the risk of complications such as infection and hypertrophic scarring. In children for whom the healing process continues beyond 8 days, there is a sigmoidal relationship between time to re‐epithelialization and the incidence of scarring, with the risk of hypertrophic scar formation increasing significantly with each additional day without epithelial closure^5^. Even incremental improvements in re‐epithelialization therefore hold promise for minimizing the physical, psychosocial and financial burdens of scarring^8^, ^34^.

The trial results build on past experimental^9^ and prospective human^10^, ^11^, ^35^, ^36^, ^37^ studies that suggested NPWT to be beneficial in the treatment of thermal injury. In a previous RCT^17^ involving 64 children with deep dermal partial‐thickness burns, NPWT reduced the duration of wound closure, bacterial colonization, skin grafting and scar severity. However, participants in the control group were treated with silver sulphadiazine cream. The silver fabric dressings employed in the present study are generally preferred by paediatric burn services because of their proven superiority in dressing change frequency, re‐epithelialization and procedural pain^27^, ^38^, ^39^. Another RCT^15^, which focused on burns affecting a larger TBSA in 50 mainly adult patients, showed that NPWT improved histopathological and biochemical markers of healing by 10 days after injury. These findings were not reported alongside any clinical outcomes. In a similar cohort of 45 adults^16^, NPWT decreased bacterial counts, wound surface area and duration of hospital stay.

Several hypotheses exist regarding the mechanism by which NPWT contributes to wound healing^40^. Its known actions include reduction in tissue oedema, maintenance of a moist environment, and induction, through microdeformational forces and localized hypoxia, of an anti‐inflammatory phenotype that promotes granulation tissue synthesis, neovascularization and remodelling of the extracellular matrix^41^. Owing to its procirculatory effects, NPWT has been considered a promising tool for attenuating burn wound progression^42^ if applied within the 48‐h interval after injury in which progressive vascular compromise is known to continue^10^, ^43^. The present trial, which set an eligibility window of 7 days, was not able to ascertain definitively whether NPWT was more efficacious if administered within this time frame. It appears likely, based on evidence from this study and others^9^, that patients might benefit from early administration, but further research is required.

The finding that NPWT produced only a moderate increase in perfusion that was not statistically significant must be interpreted with caution as there were missing data. LDI measures of mean perfusion were obtained from less than half of participants at both of the first two visits. The challenges of performing LDI in children have been well documented^28^. In previous trials^10^, ^11^, ^15^, ^35^, NPWT had positive effects on blood flow and histological neovascularization, as well as the more clinically relevant indicator of advanced depth, the need for skin grafting. As only five patients in the present study underwent grafting, the impact of NPWT on surgical requirements could not be determined precisely.

Pain represents a common concern surrounding the use of NPWT in children^44^. Encouragingly, the results showed that procedures involving NPWT were not perceived by clinicians, caregivers or children as significantly more painful than standard dressing changes. Effective pharmacological analgesia could be one plausible explanation for the low levels of pain reported across the two groups. Clinicians also took deliberate action to curb the most distressing aspects of NPWT, such as applying Niltac™ (ConvaTec, Greenlane, New Zealand) to ease extraction of the adhesive film.

The 3‐ and 6‐month follow‐up results demonstrated modest improvements in scar outcomes with NPWT. There was a statistically significant reduction in only one measure, the difference in relative scar thickness at 3 months. Whether these assessments provided an accurate representation of hypertrophic scar formation in the cohort, however, is uncertain given several limiting factors. As with previous paediatric burn studies^45^, the trial recorded low rates of long‐term follow‐up: only 15 per cent of participants attended both appointments. It also bears emphasis that the study predominantly involved superficial partial‐thickness burns with a TBSA of under 2 per cent, which are not in the highest risk category for hypertrophic scarring^6^. Additional studies are needed to determine whether the beneficial effects of NPWT might translate to a more meaningful impact on scarring in children with larger, deeper burns.

The disadvantages of NPWT warrant consideration. Six children experienced minor periwound blistering or maceration, although these incidents did not appear to impair recovery. Of greater concern were the practical challenges. Clinical management of the NPWT apparatus was more laborious than that of standard dressings, with the creation and removal of an airtight seal often requiring the most time and effort. Some caregivers struggled with technical issues that led them to seek out‐of‐hours assistance, including visits to the emergency department. Air leaks in particular were a recurrent problem, especially in burns involving joints. Finally, the size and weight (1·2 kg) of the device rendered it physically onerous to very young patients. It is possible that ultraportable NPWT systems^46^, ^47^ could provide the same therapeutic benefit with reduced burden.

A major strength of the present trial was its collection of validated patient‐centred endpoints. Many previous RCTs investigating NPWT^15^, ^16^, ^17^ based their findings on surrogate outcomes, which tend to overestimate treatment effects^14^.

In addition to the limitations already described, the 3–5‐day intervals between visits restricted the precision of the re‐epithelialization assessments. The nature of the intervention also precluded blinding of participants and clinicians, although the primary outcome was assessed by blinded review. Finally, despite eligibility criteria designed to be broadly inclusive, 57·9 per cent of patients who presented during the study period were ruled to be ineligible, principally owing to insufficient injury severity. Eligibility assessments may have been influenced by clinicians' perceptions of treatment burden, possibly constraining the generalizability of the trial's findings.

## Supporting information


**Table S1** Assessments of pain and itch
**Table S2** Scar severity*
**Table S3** Burn‐specific health‐related quality of life*: Caregivers of children <8 years
**Table S4** Burn‐specific health‐related quality of life*: Caregivers of children ≥8 years
**Table S5** Objective measures of scar severity (thickness* and colour^†^)
