# Supplementary material for: Randomized clinical trial of negative pressure wound therapy as an adjunctive treatment for small‐area thermal burns in children
Source: Br J Surg. 2020 Sep 14;107(13):1741–50. doi: 10.1002/bjs.11993 (PMC7692881; doi:10.1002/bjs.11993)
Supplement: Supplementary file 1 — Table S1 Assessments of pain and itch Table S2 Scar severity* Table S3 Burn‐specific health‐related quality of life*: Caregivers of children <8 years Table S4 Burn‐specific health‐related quality of life*: Caregivers of children ≥8 years Table S5 Objective measures of scar severity (thickness* and colour†) [file BJS-107-1741-s001.docx]

**BJS11993**

**Randomized clinical trial of negative pressure wound therapy as an adjunctive treatment for small-area thermal burns in children**

C. C. Frear, L. Cuttle, S. M. McPhail, M. D. Chatfield, R. M. Kimble and B. R. Griffin

**Table S1** Assessments of pain and itch

|  |  | | Control  Median (IQR), *n* | Intervention  Median (IQR), *n* |
| --- | --- | --- | --- | --- |
| Initial visit | **Prior to dressing change** | **FLACC scale** | 0 (0-0), 56 | 0 (0-0), 44 |
|  |  | **Observational NRS** | 0 (0-3), 55 | 1 (0-3), 47 |
|  |  | **Self-reported NRS** | 0.5 (0-3), 18 | 2 (0-3), 16 |
|  |  | **Toronto Pediatric Itch Scale** | 0 (0-0), 38 | 0 (0-0), 33 |
|  |  | **Itch Man Scale** | 0 (0-1), 27 | 0.5 (0-2), 23 |
|  | **During dressing change** | **FLACC scale** | 2 (1-4), 55 | 2 (1-4), 44 |
|  |  | **Observational NRS** | 2 (1-6), 55 | 4 (0-7), 45 |
|  |  | **Self-reported NRS** | 1 (0-4), 18 | 4 (0.75-5), 15 |
|  | **Following dressing change** | **FLACC scale** | 0 (0-0), 56 | 0 (0-0), 44 |
|  |  | **Observational NRS** | 0 (0-1), 55 | 0 (0-3), 45 |
|  |  | **Self-Reported NRS** | 0 (0-2), 18 | 2.5 (0-4.25), 15 |
|  |  | **Toronto Pediatric Itch Scale** | 0 (0-0), 38 | 0 (0-0), 32 |
|  |  | **Itch Man Scale** | 0 (0-0.25), 26 | 0 (0-1), 21 |
| At home | **Following initial visit** | **Observational NRS** | 1 (0-3), 45 | 1 (0-3), 35 |
|  |  | **Self-Reported NRS** | 1 (0-3), 17 | 3 (1-3), 11 |
|  |  | **Toronto Pediatric Itch Scale** | 0 (0-1), 26 | 0 (0-1), 22 |
|  |  | **Itch Man Scale** | 1 (1-2), 25 | 2 (1-3), 16 |
| Second visit | **Prior to dressing change** | **FLACC scale** | 0 (0-0), 54 | 0 (0-0), 46 |
|  |  | **Observational NRS** | 0 (0-1), 53 | 0 (0-1), 46 |
|  |  | **Self-reported NRS** | 2 (0-5), 19 | 3 (1.75-6), 16 |
|  |  | **Toronto Pediatric Itch Scale** | 1 (0-1), 35 | 0 (0-2), 30 |
|  |  | **Itch Man Scale** | 1 (0.25-2), 28 | 2 (0-2.25), 23 |
|  | **During dressing change** | **FLACC scale** | 2 (0-3), 53 | 2 (0-3), 45 |
|  |  | **Observational NRS** | 2 (0-6.75), 52 | 2 (0-6), 44 |
|  |  | **Self-reported NRS** | 2 (0-5), 18 | 3 (1.75-6), 15 |
|  | **Following dressing change** | **FLACC scale** | 2 (0-3), 53 | 2 (0-3), 45 |
|  |  | **Observational NRS** | 2 (0-6.75), 52 | 2 (0-6), 44 |
|  |  | **Self-Reported NRS** | 2 (0-5), 18 | 3 (1.75-6), 15 |
|  |  | **Toronto Pediatric Itch Scale** | 0 (0-0), 34 | 0 (0-0), 29 |
|  |  | **Itch Man Scale** | 0 (0-1.25), 26 | 0 (0-1.75), 21 |
| At home | **Following second visit** | **Observational NRS** | 0 (0-2), 30 | 1 (0-1.75), 20 |
|  |  | **Self-Reported NRS** | 2 (0-4.5), 9 | 1 (0-2), 8 |
|  |  | **Toronto Pediatric Itch Scale** | 1 (0-1), 21 | 0 (0-1), 11 |
|  |  | **Itch Man Scale** | 1 (1-2), 15 | 1.5 (1-3), 10 |

FLACC, Face, Legs, Activity, Cry, Consolability

NRS, Numerical Rating Scale

**Table S2** Scar severity*

|  | Control  Median (IQR) | NPWT  Median (IQR) | *P*-value^†^ |
| --- | --- | --- | --- |
| 3 months | **N=37** | **N=30** |  |
| Pain | 1 (1-1) | 1 (1-1) | 0.881 |
| Itch | 1 (1-1) | 1 (1-1) | 0.898 |
| Colour | 4 (2-6.5) | 2.5 (1-5) | 0.079 |
| Stiffness | 1 (1-3) | 1 (1-2) | 0.386 |
| Thickness | 1 (1-2.5) | 1 (1-2) | 0.768 |
| Irregularity | 2 (1-4) | 2 (1-2) | 0.365 |
| Overall opinion | 3 (1-5) | 2 (1-3) | 0.168 |
| Total score^‡^ | 17.41 (10.78) | 13.87 (8.11) | 0.142^§^ |
| 6 months | **N=31** | **N=27** |  |
| Pain | 1 (1-1) | 1 (1-1) | 0.126 |
| Itch | 1 (1-1) | 1 (1-1) | 0.575 |
| Colour | 3 (1-5) | 2 (1-4) | 0.328 |
| Stiffness | 1 (1-2) | 1 (1-1) | 0.418 |
| Thickness | 1 (1-2) | 1 (1-1) | 0.236 |
| Irregularity | 1 (1-3) | 1 (1-2) | 0.278 |
| Overall opinion | 2 (1-4) | 1 (1-2) | 0.165 |
| Total score^‡^ | 13.81 (8.67) | 9.26 (8.15) | 0.553^§^ |

*Patient component of Patient and Observer Scar Assessment Scale (POSAS)

^†^Mann-Whitney *U* test unless indicated otherwise

^‡^Values are mean (SD)

§Student’s *t*-test

**Table S3** Burn-specific health-related quality of life*: Caregivers of children <8 years

|  | Control  Median (IQR) | NPWT  Median (IQR) | *P*-value^†^ |
| --- | --- | --- | --- |
| 3 months | **N=21** | **N=15** |  |
| Overall impact | 1 (1-1.19) | 1.13 (1-1.25) | 0.409 |
| Sensory frequency | 1 (1-1) | 1 (1-1.33) | 0.216 |
| Mobility | 1 (1-1) | 1 (1-1) | 0.398 |
| Daily living | 1 (1-1) | 1 (1-1) | 0.349 |
| Friendships and social interaction | 1 (1-1) | 1 (1-1) | 1.000 |
| Appearance | 1 (1-1.33) | 1 (1-1) | 0.842 |
| Emotional reactions | 1 (1-1) | 1 (1-1) | 0.445 |
| Physical symptoms | 1.14 (1.14-1.43) | 1.14 (1-1.43) | 0.881 |
| Parent worry | 1 (1-1.33) | 1 (1-1.33) | 0.642 |
| Parent impact | 1 (1-1) | 1 (1-1) | 0.366 |
| Sensory sensitivity^‡^ | 0 (0-0.5) | 0 (0-0) | 0.912 |
| Impact on child’s daily routine^‡^ | 1 (1-1) | 1 (1-1) | 0.612 |
| Impact on developing new skills or becoming more independent^‡^ | 1 (1-1) | 1 (1-1) | 1.000 |
| Child bothered by the appearance of their scars? ^‡^ | 1 (1-1) | 1 (1-1) | 0.247 |
| 6 months | **N=14** | **N=16** |  |
| Overall impact | 1 (1-1.28) | 1 (1-1.13) | 0.823 |
| Sensory frequency | 1 (1-1.17) | 1 (1-1) | 0.205 |
| Mobility | 1 (1-1) | 1 (1-1) | 0.350 |
| Daily living | 1 (1-1) | 1 (1-1) | 0.962 |
| Friendships and social interaction | 1 (1-1) | 1 (1-1) | 0.350 |
| Appearance | 1 (1-1.67) | 1 (1-1) | 0.269 |
| Emotional reactions | 1 (1-1) | 1 (1-1) | 0.523 |
| Physical symptoms | 1.29 (1.11-1.57) | 1 (1-1.25) | 0.077 |
| Parent worry | 1 (1-1.33) | 1 (1-1) | 0.256 |
| Parent impact | 1 (1-1) | 1 (1-1) | 0.350 |
| Sensory sensitivity^‡^ | 0 (0-1) | 0 (0-0) | 0.694 |
| Impact on child’s daily routine^‡^ | 1 (1-1) | 1 (1-1) | 0.350 |
| Impact on developing new skills or becoming more independent^‡^ | 1 (1-1) | 1 (1-1) | 0.350 |
| Child bothered by the appearance of their scars? ^‡^ | 1 (1-1) | 1 (1-1) | 0.962 |

* Brisbane Burn Scar Impact Profile (BBSIP): total score for item groups were examined unless otherwise indicated

^†^ Mann-Whitney *U* test

^‡^ Individual item

**Table S4** Burn-specific health-related quality of life*: Caregivers of children ≥8 years

|  | Control  Median (IQR) | NPWT  Median (IQR) | *P*-value^†^ |
| --- | --- | --- | --- |
| 3 months | **N=8** | **N=6** |  |
| Overall impact | 1.06 (1-1.25) | 1 (1-1.22) | 0.662 |
| Sensory frequency | 1 (1-1) | 1 (1-1.25) | 0.852 |
| Mobility | 1 (1-1) | 1 (1-1) | 1.000 |
| Daily living | 1 (1-1.07) | 1 (1-1) | 0.491 |
| Friendships and social interaction | 1 (1-1) | 1 (1-1) | 0.755 |
| Appearance | 1 (1-1) | 1 (1-1.13) | 0.662 |
| Emotional reactions | 1 (1-1) | 1 (1-1.06) | 0.622 |
| Physical symptoms | 1.14 (1-1.29) | 1.14 (1.07-1.43) | 0.622 |
| Parent worry | 1 (1-1.25) | 1 (1-1.17) | 0.833 |
| Parent impact | 1 (1-1) | 1 (1-1) | 1.000 |
| Sensory sensitivity^‡^ | 0 (0-0) | 0 (0-0) | 0.755 |
| 6 months | **N=11** | **N=9** |  |
| Overall impact | 1 (1-1.25) | 1 (1-1.19) | 0.721 |
| Sensory frequency | 1 (1-1) | 1 (1-1) | 0.625 |
| Mobility | 1 (1-1) | 1 (1-1) | 1.000 |
| Daily living | 1 (1-1) | 1 (1-1) | 0.394 |
| Friendships and social interaction | 1.02 (0.08) | 1 (1-1) | 0.394 |
| Appearance | 1 (1-1) | 1 (1-1) | 0.425 |
| Emotional reactions | 1 (1-1) | 1 (1-1) | 0.366 |
| Physical symptoms | 1.14 (1-1.14) | 1.14 (1.07-1.21) | 0.369 |
| Parent worry | 1 (1-1) | 1.22 (0.67), 1 (1-1) | 0.827 |
| Parent impact | 1 (1-1) | 1.04 (0.13), 1 (1-1) | 0.269 |
| Sensory sensitivity^‡^ | 0 (0-1) | 0 (0-0) | 0.050 |

* Brisbane Burn Scar Impact Profile (BBSIP): total score for item groups were examined unless otherwise indicated

^†^ Mann-Whitney *U* test

^‡^ Individual item

**Table S5** Objective measures of scar severity (thickness* and colour^†^)

|  | Time point | Absolute difference between burn site and unaffected skin | | | *P*-value^‡^ | | Relative difference between burn site and unaffected skin^§^ | | | | *P*-value^\|^ |
| --- | --- | --- | --- | --- | --- | --- | --- | --- | --- | --- | --- |
|  |  | **Control** | **NPWT** | |  | | **Control** | | **NPWT** | |  |
|  |  | **Median (IQR), n** | |  | | **Median (IQR), n** | | | |  | |
| Scar/skin thickness (cm) | 3 months | 0.03 (0.02-0.05), 13 | 0.01 (0.01-0.02), 11 | | - | | 0.20 (0.11-0.36), 13 | | 0.08 (0.04-0.16), 11 | | 0.018 |
|  | 6 months^¶^ | 0.02 (0.01-0.03), 12 | 0.02 (0.01-0.07), 11 | | - | | 0.12 (0.04-0.39), 12 | | 0.11 (0.06-0.26), 11 | | 0.928 |
|  |  | **Mean (SD), n** | |  | |  | |  | |  | |
| Pigmentation L | 3 months | 4.89 (5.34), 15 | 5.11 (3.94), 13 | | 0.903 | | - | | - | | - |
|  | 6 months | 4.50 (3.05), 12 | 3.17 (3.11), 10 | | 0.323 | | - | | - | | - |
| Erythema a | 3 months | 2.51 (2.05), 15 | 3.64 (4.24), 13 | | 0.367 | | - | | - | | - |
|  | 6 months | 2.83 (3.17), 12 | 2.84 (3.01), 10 | | 0.997 | | - | | - | | - |

* BT12 Venue 40 MSK ultrasound machine (General Electric, Little Chalfont, UK)

^†^ DSMII ColorMeter^®^ (Cortex Technology, Hadsund, Denmark)

^‡^ Student’s *t* test

^§^ Absolute difference in thickness between burn site and unaffected contralateral side divided by total thickness of contralateral side

^|^ Mann-Whitney *U* test

^¶^Unlike the participants examined at 3 months, the children who attended the 6-month follow-up did not exhibit a statistically significant between-group difference in time to re-epithelialization (*P=*0.091)
